# Supplementary material for: Who has never tested for HIV following a community-based distribution of HIV self-test kits? Establishing associated predictors in rural Zimbabwe
Source: PLOS Glob Public Health. 2025 Apr 30;5(4):e0004459. doi: 10.1371/journal.pgph.0004459 (PMC12043167; doi:10.1371/journal.pgph.0004459)
Supplement: S1 Table — (DOCX) [file pgph.0004459.s001.docx]

**Supplementary**

**S1 Table: Explanatory variables and their levels.**

| Variable | Levels |
| --- | --- |
| Age group | *16-24 years, 25-34 years, 35-44 years, At least 45 years* |
| Sex | *Male, Female* |
| Household head Status | *Household head, Household head rep, Neither household nor head rep* |
| Level of education | *Primary complete or less , Some secondary, Secondary complete/tertiary* |
| Employment status | *Not employed, Self employed/subsistence farmer, Formally employed* |
| Religion | *Apostolic, Catholic & Protestant, Pentecostal, No religion & African tradition religion (ATR), Moslem & Other* |
| Marital status | *1st marriage/staying as married, Remarried after divorce/widowed, previously married (widowed/separated/Divorced), never married* |
| Current steady partner | *Yes, No* |
| Perceived  health status | *Very good, Good, Fair , Poor* |
| Wealth quintile | *Lowest, Second, Middle, Fourth, Highest* |
| Number of decisions (*major household purchases, about visits to your family/relatives, health care services*) participated in | *All three, One or two, None* |
| Engaged in condomless sex in the past 3 months | *Yes, No* |
| Community cohesion (critical consciousness, shared concern, social cohesion) | *Low, Medium, High* |
| Any perceived stigma in community | *Low, Medium, High* |
| Stigma: Any negative attitude | *Low, Medium, High* |
| Attitude and treatment optimism related to antiviral therapy (ART) | *Low, Medium, High* |
