# Supplementary material for: Who has never tested for HIV following a community-based distribution of HIV self-test kits? Establishing associated predictors in rural Zimbabwe
Source: PLOS Glob Public Health. 2025 Apr 30;5(4):e0004459. doi: 10.1371/journal.pgph.0004459 (PMC12043167; doi:10.1371/journal.pgph.0004459)
Supplement: S2 Table — (DOCX) [file pgph.0004459.s003.docx]

**S2 Table: Derivation of constructs**

| **Subscales** | **Items / statements** | **How the variables were derived** | **Cronbach’s alpha (items)** | **KMO Measure of Sampling Adequacy** | **Factor loadings (pattern matrix) and unique variances** | **Number of eigen values>1**  ***(Proportion variance explained)*** |
| --- | --- | --- | --- | --- | --- | --- |
| **Community cohesion** | | | | | | |
| Social cohesion  (6 items) | - People in this village are willing to help their neighbours (cm04). - This is a close-knit community(cm05). - People in this village can be trusted(cm06). - People in this village generally get along well with each other(cm07). - People in this village share the same values(cm08). - People in this village look out for each other(cm09). | - Factor analysis (principal component factoring method) with one factor solution was conducted. - Summarised as the median value in cluster for a cluster level summary - Grouped clusters into high/middle/low based on the calculated median. | 0.88 | 0.88 | \| Variable \| Factor1 \| Uniqueness \| \| --- \| --- \| --- \| \| m04 \| 0.74 \| 0.46 \| \| cm05 \| 0.83 \| 0.31 \| \| cm06 \| 0.86 \| 0.26 \| \| cm07 \| 0.86 \| 0.26 \| \| cm08 \| 0.67 \| 0.55 \| \| m09 \| 0.81 \| 0.34 \| | 1(0.64) |
| Shared concern  (10 items) | - People in your village are concerned about HIV(cm10). - People in your village consider HIV/AIDS an important issue(cm11). - People in your village talk openly about HIV(cm12). - People in your village believe that HIV impacts the community(cm13). - People in your village talk about HIV/AIDS at community meetings(cm14). - People in your village work together to prevent HIV from spreading(cm15). - People in your village work together to reduce the effects of HIV(cm16). - People in your village believe they can change the course of the HIV/AIDS epidemic(cm17). - People in your village exchange information about HIV/AIDS(cm18). - People in your village take HIV/AIDS seriously(cm19). | - Factor analysis (principal component factoring method) with one factor solution was conducted. - Summarised as the median value in cluster for a cluster level summary. - Grouped clusters into high/middle/low based on the calculated median. | 0.92 | 0.93 | \| Variable \| Factor1 \| Uniqueness \| \| --- \| --- \| --- \| \| cm10 \| 0.74 \| 0.46 \| \| cm11 \| 0.77 \| 0.41 \| \| cm12 \| 0.77 \| 0.41 \| \| cm13 \| 0.63 \| 0.60 \| \| cm14 \| 0.71 \| 0.49 \| \| cm15 \| 0.82 \| 0.33 \| \| cm16 \| 0.83 \| 0.32 \| \| cm17 \| 0.76 \| 0.43 \| \| cm18 \| 0.75 \| 0.43 \| \| cm19 \| 0.80 \| 0.35 \| | 1(0.58) |
| Critical consciousness  (11 items) | - People work together to solve problems in the village (cm20). - People in your village talk to each other about how to solve village problems(cm21). - People in your village enjoy discussing different ways to solve village problems(cm22). - People in your village are open to hearing different views about community problems and solutions(cm23). - People in your village volunteer to help solve village problems(cm24). - People in your village think about why there are problems so they can address the cause of problems(cm25). - There is a lot of cooperation between groups in the village(cm26). - People in this village not only talk about problems but they also try to solve them(cm27). - If your community fails to resolve a community problem, they will try another-different approach to solve the problem(cm28). - If your community fails to resolve a community problem, they will learn from that experience and do a better job when they try to solve the problem in the future(cm29). - If leaders in the village fail to resolve a village problem, the villagers will work together to find a solution(cm30). | - Factor analysis (principal component factoring method) with one factor solution was conducted. - Summarised as the median value in cluster for a cluster level summary. - Grouped clusters into high/middle/low based on the calculated median. | 0.95 | 0.96 | \| Variable \| Factor1 \| Uniqueness \| \| --- \| --- \| --- \| \| cm20 \| 0.78 \| 0.39 \| \| cm21 \| 0.82 \| 0.33 \| \| cm22 \| 0.83 \| 0.31 \| \| cm23 \| 0.82 \| 0.33 \| \| cm24 \| 0.85 \| 0.28 \| \| cm25 \| 0.83 \| 0.32 \| \| cm26 \| 0.78 \| 0.39 \| \| cm27 \| 0.82 \| 0.32 \| \| cm28 \| 0.81 \| 0.35 \| \| cm29 \| 0.79 \| 0.38 \| \| cm30 \| 0.78 \| 0.38 \| | 1(0.66) |
| **Stigma** | | | | | | |
| Any negative attitude  (3 items) | - I would be ashamed if someone in my family had HIV(h08) - I would not like to sit close to someone living with HIV, for example on public transport, at church, or in a waiting room(h09) - I fear that I could contract HIV if I come into contact with the saliva of a person with HIV(h10) | - Factor analysis (principal component factoring method) with one factor solution was conducted. - Factor scores were categorised into high/middle/low. | 0.78 | 0.69 | \| Variable \| Factor1 \| Uniqueness \| \| --- \| --- \| --- \| \| h08 \| 0.84 \| 0.30 \| \| h09 \| 0.86 \| 0.25 \| \| h10 \| 0.81 \| 0.35 \| | 1(0.70) |
| Any perceived stigma in community  (5 items) | - People sometimes talk badly about people living with or thought to be living with HIV(h03) - People living with or thought to be living with HIV lose respect or standing (h05). - People living with or thought to be living with HIV are verbally insulted, harassed, and/or threatened(h06). - People living with or thought to be living with HIV are sometimes physically assaulted (h07). - People sometimes disclose that other people are HIV positive without their permission (h11) | - Factor analysis (principal component factoring method) with one factor solution was conducted. - Summarised as the median value in cluster for a cluster level summary. - Grouped clusters into high/middle/low based on the calculated median. | 0.78 | 0.75 | \| Variable \| Factor1 \| Uniqueness \| \| --- \| --- \| --- \| \| h03 \| 0.55 \| 0.70 \| \| h05 \| 0.82 \| 0.34 \| \| h06 \| 0.85 \| 0.27 \| \| h07 \| 0.77 \| 0.40 \| \| h11 \| 0.62 \| 0.62 \| | 1(0.53) |
| **Attitudes and treatment optimism related to Antiretroviral Therapy (ART)** | | | | | | |
| Attitudes and treatment optimism to HIV treatment (4 items) | - I would feel safe having sex with someone who is HIV-positive as long as they are receiving HIV treatment (p03). - I am less worried about HIV infection than I used to be (p04). - HIV treatment makes me less anxious about having unprotected sex (p05). - HIV treatment can help prevent a person with HIV from infecting a partner (p09). | - Factor analysis (principal component factoring method) with one factor solution was conducted. - Factor scores categorised into high/middle/low. | 0.75 | 0.75 | \| Variable \| Factor1 \| Uniqueness \| \| --- \| --- \| --- \| \| p03 \| 0.59 \| 0.65 \| \| p04 \| 0.80 \| 0.36 \| \| p05 \| 0.83 \| 0.32 \| \| p09 \| 0.80 \| 0.37 \| | 1(0.58) |

** **P values for Bartlett test of sphericity for all the subscales were below 0.001**
