# Supplementary material for: Who has never tested for HIV following a community-based distribution of HIV self-test kits? Establishing associated predictors in rural Zimbabwe
Source: PLOS Glob Public Health. 2025 Apr 30;5(4):e0004459. doi: 10.1371/journal.pgph.0004459 (PMC12043167; doi:10.1371/journal.pgph.0004459)
Supplement: S3 Table — (DOCX) [file pgph.0004459.s004.docx]

**S3 Table: Unadjusted and adjusted analysis of risk factors associated with never having tested for HIV.**

|  | **Unadjusted OR** | **95% CI]** | **P Value** | **Adjusted OR**  **(Model I)^a^** | **95% CI** | **P Value** | **Adjusted OR**  **(Model II)^b^** | **95% CI** | **P Value** |
| --- | --- | --- | --- | --- | --- | --- | --- | --- | --- |
| **Population Characteristics** |  |  |  |  |  |  |  |  |  |
| **Age** |  |  | <0.001^c^ |  |  | <0.001^c^ |  |  | <0.001 ^c^ |
| 16-24 years | 3.89 | [3.28, 4.61] |  | 3.84 | [3.23 , 4.55] |  | 3.93 | [3.31 , 4.68] |  |
| 25-34 years | 1.29 | [1.06, 1.57] |  | 1.30 | [1.07, 1.59] |  | 1.32 | [1.09 , 1.61] |  |
| 35-44 years | 1.0 | ref |  | 1.0 | ref |  | 1.0 | ref |  |
| 45 + years | 2.15 | [1.79, 2.58] |  | 2.17 | [1.80 , 2.60] |  | 1.97 | [1.63, 2.37] |  |
| **Sex** |  |  | <0.001 |  |  | <0.001 |  |  | <0.001 |
| Female | 1.0 | ref |  | `1.0 | ref |  | 1.0 | ref |  |
| Male | 1.74 | [1.57, 1.93] |  | 1.69 | [1.52 , 1.87] |  | 1.66 | [1.48, 1.85] |  |
| **Household Head status:** |  |  | <0.001^c^ |  |  | <0.001^c^ |  |  | <0.001^c^ |
| Household head | 1.0 | ref |  | 1.0 | ref |  | 1.0 | ref |  |
| Household head rep | 0.74 | [0.61, 0.91] |  | 0.80 | [0.64, 1.00] |  | 0.79 | [0.64, 0.99] |  |
| Not household head/rep | 1.62 | [1.45, 1.80] |  | 1.38 | [1.21, 1.57] |  | 1.36 | [1.20, 1.55] |  |
| **Education** |  |  | <0.001^c^ |  |  | <0.001^c^ |  |  | <0.001^c^ |
| Primary Complete/less | 1.35 | [1.19, 1.53] |  | 1.68 | [1.46, 1.92] |  | 1.64 | [1.43 , 1.88] |  |
| Some Secondary | 1.67 | [1.46, 1.90] |  | 1.62 | [1.42, 1.86] |  | 1.61 | [1.41 , 1.85] |  |
| Secondary Complete/tertiary | 1.0 | ref |  | 1.0 | ref |  | 1.0 | ref |  |
| **Employment** |  |  | <0.001^c^ |  |  | <0.001^c^ |  |  | <0.001^c^ |
| Formally employed | 1.0 | ref |  | 1.0 | ref |  | 1.0 | ref |  |
| Self employed/subsistence farmer | 1.10 | [0.86, 1.40] |  | 0.95 | [0.74, 1.21] |  | 0.88 | [0.68, 1.12] |  |
| Unemployed | 1.45 | [1.21, 1.75] |  | 1.39 | [1.15, 1.69] |  | 1.30 | [1.07, 1.57] |  |
| **Religion:** |  |  | <0.001^c^ |  |  | <0.001^c^ |  |  | <0.001^c^ |
| Apostolic | 1.0 | ref |  | 1.0 | ref |  | 1.0 | ref |  |
| Catholic/ Protestant | 1.30 | [1.13, 1.49] |  | 1.15 | [1.00, 1.32] |  | 1.21 | [1.04 , 1.39] |  |
| Pentecostal | 0.89 | [0.74, 1.08] |  | 0.87 | [0.72, 1.05] |  | 0.94 | [0.78 , 1.14] |  |
| No religion/ ATR | 1.60 | [1.38, 1.85] |  | 1.41 | [1.20, 1.64] |  | 1.35 | [1.15 , 1.57] |  |
| Moslem & Other | 1.35 | [1.14, 1.60] |  | 1.25 | [1.04, 1.48] |  | 1.28 | [1.07 , 1.53] |  |
| **Marital Status:** |  |  | <0.001^c^ |  |  | <0.001^c^ |  |  | <0.001^c^ |
| First marriage | 1.0 | ref |  | 1.0 | ref |  | 1.0 | ref |  |
| Remarried after divorce/widowed | 0.85 | [0.70, 1.04] |  | 0.85 | [0.70, 1.05] |  | 0.82 | [0.67, 1.00] |  |
| Widowed/separated/Divorced | 1.47 | [1.25, 1.73] |  | 1.41 | [1.19, 1.68] |  | 1.33 | [1.12, 1.59] |  |
| Never married | 4.14 | [3.66, 4.68] |  | 3.48 | [2.98, 4.07] |  | 3.54 | [3.03, 4.15] |  |
| **Current steady partner** |  |  |  |  |  |  |  |  |  |
| Yes | 1.0 | ref |  | 1.0 | ref |  | 1.0 | ref |  |
| No | 3.25 | [2.93, 3.62] | <0.001 | 2.68 | [2.40, 2.99] | <0.001 | 2.60 | [2.33, 2.91] | <0.001 |
| **Perceived health status:** |  |  | <0.001^c^ |  |  | 0.111^c^ |  |  | 0.058 ^c^ |
| Very good | 1.0 | ref |  | 1.0 | ref |  | 1.0 | ref |  |
| Good | 0.95 | [0.84, 1.08] |  | 1.12 | [0.98, 1.28] |  | 1.10 | [0.97, 1.26] |  |
| Fair | 0.76 | [0.66, 0.87] |  | 0.99 | [0.86, 1.15] |  | 0.98 | [0.84, 1.13] |  |
| Poor | 0.64 | [0.52, 0.77] |  | 0.92 | [0.75, 1.12] |  | 0.86 | [0.70, 1.05] |  |
| **Wealth quintile:** |  |  | 0.028^c^  0.884^d^ |  |  | 0.016^c^  0.687^d^ |  |  | 0.016^c^  0.264^d^ |
| Highest | 1.0 | ref |  | 1.0 | ref |  | 1.0 | ref |  |
| Lowest | 1.06 | [0.89, 1.25] |  | 1.12 | [0.94, 1.33] |  | 0.99 | [0.83, 1.18] |  |
| Second | 0.81 | [0.69, 0.96] |  | 0.84 | [0.70, 0.99] |  | 0.77 | [0.65, 0.92] |  |
| Middle | 1.00 | [0.85, 1.18] |  | 1.02 | [0.87, 1.21] |  | 0.95 | [0.81, 1.12] |  |
| Fourth | 0.91 | [0.78, 1.07] |  | 0.90 | [0.77, 1.06] |  | 0.86 | [0.73, 1.01] |  |
| **Number of decisions participated in** |  |  | <0.001^c^ |  |  | <0.001^c^ |  |  | <0.001^c^ |
| Three | 1.0 | ref |  | 1.0 | ref |  | 1.0 | ref |  |
| One/ two | 1.18 | [1.04 ,1.33] |  | 0.97 | [0.85 , 1.09] |  | 0.96 | [0.84 , 1.09] |  |
| None | 3.04 | [2.67 , 3.46] |  | 2.07 | [1.79 , 2.39] |  | 1.95 | [1.69 , 2.25] |  |
|  |  |  |  |  |  |  |  |  |  |
| **Condomless sex in the past 3 months** |  |  | <0.001 |  |  | <0.001 |  |  | <0.001 |
| Yes | 1.0 | ref |  | 1.0 | ref |  | 1.0 | ref |  |
| No | 3.07 | [2.76 , 3.41] |  | 2.58 | [2.31 , 2.87] |  | 2.53 | [2.27 , 2.82] |  |
| **Social cohesion:** |  |  | 0.028^c^  0.213^d^ |  |  | 0.098^c^  0.250^d^ |  |  | 0.208^c^  0.207^d^ |
| Low | 1.0 | ref |  | 1.0 | ref |  | 1.0 | ref |  |
| Medium | 1.35 | [1.06, 1.70] |  | 1.32 | [1.03, 1.68] |  | 1.27 | [0.98, 1.64] |  |
| High | 1.20 | [0.95, 1.51] |  | 1.16 | [0.91, 1.47] |  | 1.16 | [0.90, 1.50] |  |
| **Critical consciousness:** |  |  | 0.962^c^  0.788^d^ |  |  | 0.915^c^  0.966^d^ |  |  | 0.990^c^  0.678^d^ |
| Low | 1.04 | [0.81, 1.33] |  | 1.05 | [0.82, 1.35] |  | 1.01 | [0.77, 1.31] |  |
| Medium | 1.01 | [0.79, 1.29] |  | 1.02 | [0.79, 1.30] |  | 0.99 | [0.76, 1.28] |  |
| High | 1.0 | ref |  | 1.0 | ref |  | 1.0 | ref |  |
| **Shared Concern:** |  |  | 0.840^c^  0.616^d^ |  |  | 0.732^c^  0.672^d^ |  |  | 0.915^c^  0.931^d^ |
| Low | 1.07 | [0.84, 1.37] |  | 1.10 | [0.86, 1.41] |  | 1.06 | [0.81, 1.37] |  |
| Medium | 1.07 | [0.83, 1.37] |  | 1.04 | [0.81, 1.34] |  | 1.02 | [0.78, 1.33] |  |
| High | 1.0 | ref |  | 1.0 | ref |  | 1.0 | ref |  |
| **Perceived Stigma in community:** |  |  | 0.632^c^  0.438^d^ |  |  | 0.695^c^  0.669^d^ |  |  | 0.818^c^  0.604^d^ |
| Low | 1.0 | ref |  | 1.0 | ref |  | 1.0 | ref |  |
| Medium | 0.99 | [0.74, 1.31] |  | 0.94 | [0.70, 1.25] |  | 0.98 | [0.72, 1.33] |  |
| High | 1.10 | [0.81, 1.48] |  | 1.03 | [0.76, 1.40] |  | 1.05 | [0.76, 1.46] |  |
| **Stigma: Any negative attitude** |  |  | <0.001^c^  <0.001^d^ |  |  | <0.001^c^  <0.001^d^ |  |  | <0.001^c^  <0.001^d^ |
| Low | 1.0 | ref |  | 1.0 | ref |  | 1.0 | ref |  |
| Medium | 1.06 | [0.93, 1.22] |  | 1.04 | [0.90, 1.19] |  | 1.03 | [0.90, 1.18] |  |
| High | 1.63 | [1.43, 1.85] |  | 1.42 | [1.24, 1.62] |  | 1.38 | [1.20, 1.57] |  |
| **Treatment optimism related to ART** |  |  | <0.001^c^  <0.001^d^ |  |  | <0.001^c^  <0.001^d^ |  |  | <0.001^c^  <0.001^d^ |
| Low | 1.60 | [1.40, 1.83] |  | 1.52 | [1.32 ,1.74] |  | 1.56 | [1.36, 1.79] |  |
| Medium | 1.57 | [1.37, 1.79] |  | 1.53 | [1.33 , 1.75] |  | 1.56 | [1.36, 1.79] |  |
| High | 1.0 | ref |  | 1.0 | ref |  | 1.0 | ref |  |

a-Adjusted for age and sex, b- adjusted for age, sex, education and religion, c- overall p value, d -P value for trend

CI- confidence interval; OR-Odds ratio
