# Supplementary material for: Who has never tested for HIV following a community-based distribution of HIV self-test kits? Establishing associated predictors in rural Zimbabwe
Source: PLOS Glob Public Health. 2025 Apr 30;5(4):e0004459. doi: 10.1371/journal.pgph.0004459 (PMC12043167; doi:10.1371/journal.pgph.0004459)
Supplement: S4 Table — (DOCX) [file pgph.0004459.s005.docx]

**S4 Table : Descriptive characteristics of participants who reported no perception of HIV risk (n=1822)**

|  | **Perceived HIV risk** | **No perception of HIV risk** | **P value** |
| --- | --- | --- | --- |
| **Population Characteristics** |  |  |  |
| **N** | 910(49.9%) | 912 (50.1%) |  |
| **Age category** |  |  |  |
| 16-24 years | 440(49.2%) | 454(50.8%) |  |
| 25-34 years | 170(60.5%) | 111(39.5% |  |
| 35-44 years | 111(60.7%) | 72(9.4%) |  |
| 45 + years | 189(40.7%) | 275(59.3%) | <0.001 |
| **Sex** |  |  |  |
| Female | 378(48.0%) | 410(52.0%) |  |
| Male | 532 (51.5%) | 502 (48.5%) | 0.141 |
| **Household Head status:** |  |  |  |
| Household head | 310(51.6%) | 291(48.4%) |  |
| Household head rep | 61(48.0%) | 66(52.0%) |  |
| Not household head/rep | 539(49.3%) | 555(50.7%) | 0.598 |
| **Education level:** |  |  |  |
| Secondary Complete/tertiary | 299(55.7%) | 238(44.3%) |  |
| Some Secondary | 299(52.7%) | 268(43.3%) |  |
| Primary Complete/less | 312(43.4%) | 406(56.6%) | <0.001 |
| **Employment Status:** |  |  |  |
| Formally employed | 711 (48.5%) | 754 (51.5%) |  |
| Self employed/subsistence farmer | 85 (51.5%) | 80(48.5%) |  |
| Not employed | 91 (59.5%) | 62(40.5%) | 0.032 |
| **Religion:** |  |  |  |
| Apostolic | 318 (53.1%) | 281 (46.9%) |  |
| Catholic/ Protestant | 227(49.6%) | 231 (50.4%) |  |
| Pentecostal | 87(51.5%) | 82 (48.5%) |  |
| No religion/ ATR | 180 (49.0%) | 187 (51.0%) |  |
| Moslem & Other | 98 (42.8%) | 131 (57.2%) | 0.118 |
| **Marital Status:** |  |  |  |
| 1st marriage/staying as married | 288(52.0%) | 266(48.0%) |  |
| Remarried after divorce/widowed | 69(52.3%) | 63(47.7%) |  |
| Widowed/separated/Divorced | 111(46.1%) | 130 (53.9%) |  |
| Never married | 405(48.0%) | 432(52.0%) | 0.307 |
| **Current steady partner** |  |  |  |
| Yes | 399(55.0%) | 327(45.0%) |  |
| No | 478(46.0%) | 561(54.0%) | <0.001 |
| **Perceived health status:** |  |  |  |
| Very good | 252(43.6%) | 326(56.4%) |  |
| Good | 323(51.2%) | 308(48.8%) |  |
| Fair | 232(54.6%) | 193(45.4%) |  |
| Poor | 82(52.6%) | 74 (47.4%) | 0.003 |
| **Household asset quintile:** |  |  |  |
| Highest | 167(48.0%) | 154(52.0%) |  |
| Lowest | 143(50.2%) | 142(49.8%) |  |
| Second | 186(52.5%) | 168(47.5%) |  |
| Middle | 167(48.4%) | 178(51.6%) |  |
| Fourth | 191(47.2%) | 214(52.8%) | 0.541 |
| **Participation in decisions (Purchases, health care and family visits)** |  |  |  |
| All three | 407(50.4%) | 400(49.6%) |  |
| One or two | 257(51.7%) | 240(48.3%) |  |
| None | 246(47.5%) | 272(52.5%) | 0.378 |
| **Condomless sex in the past 3 months** |  |  |  |
| Yes | 366(55.9%) | 289(44.1%) |  |
| No | 544(46.6%) | 623(54.4%) | <0.001 |
| **Social cohesion:** |  |  |  |
| Low | 220(53.3%) | 193(46.7%) |  |
| Medium | 311(46.9%) | 352(53.1%) |  |
| High | 379(50.8%) | 367(49.2%) | 0.106 |
| **Critical consciousness:** |  |  |  |
| Low | 282(48.0%) | 310(52.0%) |  |
| Medium | 308(50.6%) | 301(49.4%) |  |
| High | 316(51.2%) | 301(48.8%) | 0.494 |
| **Shared Concern:** |  |  |  |
| Low | 288(47.1%) | 324(52.9%) |  |
| Medium | 317(51.0%) | 305(49.0%) |  |
| High | 305(51.9%) | 283(48.1%) | 0.205 |
| **Perceived Stigma in community:** |  |  |  |
| Low | 143(54.8%) | 118(45.2%) |  |
| medium | 464(51.7%) | 434(48.3%) |  |
| High | 303(45.7%) | 360(54.3%) | 0.016 |
| **Stigma: Any negative attitude** |  |  |  |
| Low | 217(50.5%) | 213(49.5%) |  |
| Medium | 320(53.2%) | 281(46.8%) |  |
| High | 373(47.2%) | 418(52.8%) | 0.077 |
| **Treatment optimism:** |  |  |  |
| Low | 310(49.4%) | 317(50.6%) |  |
| Medium | 328(52.4%) | 298(47.6%) |  |
| High | 197(46.0%) | 231(54.0%) | 0.126 |
