# Supplementary material for: Who has never tested for HIV following a community-based distribution of HIV self-test kits? Establishing associated predictors in rural Zimbabwe
Source: PLOS Glob Public Health. 2025 Apr 30;5(4):e0004459. doi: 10.1371/journal.pgph.0004459 (PMC12043167; doi:10.1371/journal.pgph.0004459)
Supplement: Attachment — Submitted filename: PGPH Manuscript Response to Reviewer.docx [file pgph.0004459.s009.docx]

**Manuscript PGPH-D-24-01522: 'Who has never tested for HIV following a community-based**

**distribution of HIV self-test kits? Establishing associated predictors in rural Zimbabwe**.’

Dear Dr. Tsitsi B. Masvawure.

Thank you for giving us the opportunity to submit a revised draft of the manuscript for publication in PLOS Global Public Health. We appreciate the time and effort that you and the reviewers dedicated to providing valuable feedback on our manuscript and are grateful for the insightful comments. We have incorporated most of the suggestions made by the reviewers in the manuscript and below in blue are point-by-point to the reviewers’ comments.

**Journal Requirements:**

Please include a complete copy of PLOS’ questionnaire on inclusivity in global research in your revised manuscript.

**Response:** The questionnaire was completed and uploaded as supporting information.

In the online submission form, you indicated that "Data will be available upon request". All PLOS journals now require all data underlying the findings described in their manuscript to be freely available to other researchers.

**Response:** The data was uploaded as supplementary information.

Please provide an Author Summary

Authors summary added between the abstract and the introduction as requested.

We have noticed that you have uploaded Supporting Information files, but you have not included a list of legends. Please add a full list of legends for your Supporting Information files after the references list.

**Response:** Well noted, thank you. A list of legends for supporting information files was added after the reference list.

**Additional Editor Comments:**

Dear Authors,

I would like to add that it may help if you describe the community intervention early on in the introduction or methods section, so that it is clear that data was collected from communities that had received the community HIV self-testing intervention. It took me some time to figure this out. You may also need to explain in a bit more detail how the HIV self-testing intervention was marketed and how community members were made aware of this initiative.

**Response:** Thank you for that observation, we have now added in the Methods in the section “Study design and Settings” (*line 134-164 in clean version and 147-175 in track changes version*) the following text: “Briefly, in the CRT, clusters were defined as headman units, which are administrative units in rural areas of Zimbabwe responsible for implementing community level activities. Forty clusters were randomly allocated to either community-led or paid HIVST distribution arms using 1:1 restricted randomisation. To be considered potentially eligible, clusters had to have at least three census enumeration areas (EAs) within its boundaries, not share a health facility with a neighbouring cluster and the distance between any two selected clusters was at least 20 km. Randomisation was restricted on district, distance from the centre of the headman unit to the nearest health facility and availability of PrEP at the nearest health facility. Consent for communities to participate was sought and obtained from the community leaders.

In the community-led arm, community members at least 16 years of age were invited to the first meeting during which they were introduced to the concept of a community-led model and the importance of HIV testing. The concept of antiretroviral treatment (ART) for preventing HIV transmission within their headman unit and the potential to reduce new HIV infections through early HIV testing and linkage to ART was discussed and explained. Members were entrusted with the responsibility of developing their own HIVST kits distributing models with the options of selecting distributors, deciding on incentives for distributors, managing of kits and how to ensure adherence to regulatory requirements. The distribution models had to adhere to the following MOHCC guidelines testing criteria: kits only to be given to individuals 16 years old and above (age of consent for HIV testing), no forced testing or results disclosure, and confidentiality to be upheld. HIVST distributors received a 3-day training program according to MOHCC guidelines. The training covered areas on HIV testing, facilitating utilization of HIVST kits by others, sharing information to promote and facilitates access to appropriate post-test services, providing information on effectiveness of ART for preventing HIV, and how to complete the data collection tool. Distribution of kits was done over a period of 4-6 weeks.

In the paid distributor model, distributors received the same 3-day training as in the community led model and had 4 weeks to conduct door-to-door distribution in one or two villages. PSI provided training, kits, and a one-off stipend of US$50 to distributors.

In both HIVST distribution models, distributors were expected to give kits to all willing individuals (one kit per person) in their geographical area who met the testing criteria and share information about linkage to all post-test services that testers could access from local clinics.”

Regarding the data collection, the methods (*Study population and data collection section*) already contains this sentence “Trial outcomes were evaluated using a household survey 4 months post-distribution to determine uptake of HIVST and linkage to post-test services.” *line 155-156 in clean version and 193-195 in track changes version)*

**Response to Reviewers**

**Reviewer #1: General comments**

The manuscript is well written, an interesting read and provides a unique insight into an

important topic in an exciting field of HIV self-testing delivery models in general

populations in Zimbabwe. In their analysis, authors clearly identify sub-groups within the

general populations who did not test for HIV despite the wider availability of HIV self-test

kits in the community.

A major observation is that there is limited discussion on implications of scaled distribution of HIV self-test kits in the community on the number of those who have never tested. Again, authors are not clear on specific combinations of attributes of sub-populations that are important to implementers who are intending to provide targeted distribution of HIV self-test kits in the community.

**Response:** Regarding the limited discussion on implications of scaled distribution of HIV self-test kits on the number who have never tested, previous research (Hatzold K,2019; Johnson CC, 2017) reported a positive association between HIVST distribution and testing rates. Systematic review and meta-analysis of studies conducted in Sub Saharan Africa reported that a range of HIVST distribution models were associated with an increase in reported HIV testing, thus scaled distribution of HIVST kits is likely to reduce the numbers of those who have never tested for HIV. To address the reviewer concern we have added the following text at *line 358-363 in clean version and 447-452 in track changes version*: “This study did not collect baseline information on HIV testing uptake in the communities and did not evaluate whether the introduction of community-based HIV self-testing increases testing rates. However, previous evidence had reported that HIVST increases uptake of HIV testing [32, 33]. It is also important to provide a range of HIVST delivery models, as testing rates have been shown to improve with a variety of HIVST models and support tools [34].”

We identified attributes that were associated with higher likelihood of being never testers and these were adjusted by age and sex. Because we thought a priori that the association between condomless sex and never testing could differ by gender.

**Specific comments**

**Title**

Title needs rephrasing: 'Who has never tested for HIV following a community-based

distribution of HIV self-test kits? Establishing associated predictors in rural Zimbabwe

**Response:** We rephrased the title as suggested, thanks.

**Abstract**

Line 33: Indicate the year when this was first achieved

Line 34: Rephrase: 'some groups of people...'

Line 35: Rephrase to 'we estimated the prevalence and predictors of community members

who with a 'never-having tested for HIV' status following community-based distribution of

HIV self-test kits in rural Zimbabwe.

**Response:** We added the year when the targets were first achieved (*line 31 in clean version and 34 in track changes version*) and rephrased the line *31 in clean version and 34 in track changes version*). Regarding *lines 33-35 in clean version and 36-38 in track changes version*, we have rephrased it to read as “we estimated the prevalence and predictors of 'never-having tested for HIV' status following community-based distribution of HIV self-test kits in rural Zimbabwe”.

**Introduction**

Line 64-65: The statement requires a reference

**Response:** We added reference to the statement, see *line 78 in clean version and 84 in track changes version*

Lines 65-67: Rephrase the statement 'Globally, the burden of HIV is highest within the

Eastern and Southern Africa (ESA) region which accounts for more than half (54%) of all

people living with HIV (PLHIV) in the world [1]; but the region only has 7% of the global

population [2].

**Response:** We rephrased the first part of the sentence as suggested, but left the second part as it was as we think it reads better. See l*ine 79-81 in clean version and 58-89 in track changes version*

Lines 68-68: Please rephrase: ' Although a considerable decline in HIV incidence (-44%)

and AIDS related deaths (-58%) were estimated between 2010 and 2021 in ESA compared

with other ...'

**Response:** The 44%, was a decline in number of new infections without considering the population size (incidence), so we retained the sentence with minor changes as reflected in the document *with track changes line 90-91 and 82-83 in clean version*. The new sentence reads; “Although the estimated numbers of new HIV infections has declined by 44% and estimated AIDS related deaths by 58% between 2010 and 2021…”

Lines 71-73: Kindly provide reasons why there has been such a dramatic decline in HIV

incidence and prevalence in Zimbabwe.

**Response:** We have added the following text to describe the reasons for the decline in incidence and prevalence of HIV (*line 87-95 in clean version and 96-104 in track changes version*): “The reduction in HIV incidence and prevalence was mainly due to reduction in sexual risk behaviour [8, 9], increase in number of people living with HIV on antiretrovirals[10], implementation of the Extended Zimbabwe National HIV and AIDS Strategic Plan (ZNASP III) 2015-2020[11], which aimed to achieve the 90-90-90 targets and focused on high-impact interventions for key and vulnerable populations. Key program activities included combination HIV prevention strategies, social and behaviour change communication campaigns, widespread condom promotion and distribution, the promotion of male circumcision and robust prevention of mother-to-child transmission programs.

Lines 77-78: Kindly describe these inequalities and why they exist?

Response: We provided a brief description of possible reasons for these inequalities as requested. The sentence now reads as “there are specific subpopulations, such as men[14,15] , key populations (female sex workers (FSW) and men who had sex with men (MSM))[16] and especially young people (including children), that are still lagging behind in terms of knowledge of HIV status. These inequalities are probably due to lack of innovative case finding in children [15], offer of HIV testing being primarily in clinics and discriminating laws against MSM and sex workers [16].” See line *98-102 in clean version and 109-113 in track changes version*

Lines 79-83. I suggest breaking the sentence into two parts. One part should be about

increased access to HIV testing services and the other part should be about HIV self-

testing as an additional option.

**Response:** The new sentences now read as: “In 2014 the Ministry of Health and Child Care (MOHCC) highlighted the need for increased access to HIV testing services, given the compelling HIV infection rates in the country [17]. In 2016 MOHCC then recommended HIV self-testing (HIVST) as an additional approach to HIV testing services [18] following extensive STAR consortium work on HIVST [19].” See *line 103-109 in clean version and 114-118 in track changes version*

Line 86: Add Zimbabwe to the sentence. i.e. 'The annual number of conventional HIV test

performed in Zimbabwe...'

**Response:** We added “in Zimbabwe” as suggested and the sentence now reads: “The annual number of conventional HIV tests performed in Zimbabwe increased from 1.7 million in 2011 to 3 million in 2018…” *line 110 in clean version and 121 in track changes version*

Line 88-89: It would be great to have more current estimates that are consistent with the

first 95% that Zimbabwe has achieved.

**Response:** Well noted, however, those are the most up to date estimates from the surveys conducted in the country. We are still awaiting results of the latest Zimbabwe demographic and health survey, which was conducted in 2023-24.

Lines 91-92: Is HIVST part of the conventional HIV testing as described in line 86-87?

**Response**: HIVST is not part of conventional testing. However, all positive HIV tests results (reactive tests) using HIVST should be confirmed using conventional testing. We have added the statement: “with those with reactive results being confirmed with conventional testing” after HIVST (facility- and community-based). See *line 116 in clean version and 127 in track changes version*

Line 93: There is a need for a comma between the word 'test' and 'there'

**Response:** Comma added, thank you for the input.

Lines 96-97: Kindly use past tense i.e. 'This study estimated the prevalence of never having

tested for HIV...'

**Response:** The corrected sentence is “Therefore, this study estimated the prevalence of never having tested for HIV among people living in rural areas of Zimbabwe and identify the associated characteristics.” See *line 121-123 in clean version and 132-134 in track changes version*

Lines 98-99: 'We also assessed factors associated with reporting no perceived HIV risk among participants who never tested for HIV.'

**Response:** Thank you for the correction, the sentence was changed as recommended. See *line 123-124 in clean version and 134-135 in track changes version*

**Methods**

Line 103: Rephrase the sentence 'The study analysed data from a cluster randomised

trial...'

**Response:** We rephrased the sentence from “The study uses data…..” to “The study analysed data…..”

Line 122: ...did not share a health facility with a neighboring cluster...

**Response:** We rephrased the sentence to; “To be considered potentially eligible, clusters had to have at least three census enumeration areas (EAs) within its boundaries, did not share a health facility with a neighbouring cluster and the distance between any two selected clusters was at least 20 km”. Thank you. *line 137-140 in clean version and 150-153 in track changes version*

Lines 125-128: The sentence is incoherent. Please review and revise it

**Response:** The sentence was revised to: “All eligible individuals (16 years and above) residing in the selected enumeration areas during HIVST distribution, upon giving written consent to participate in the survey, completed a self-administered Audio Computer-Assisted Survey Instrument questionnaire (ACASI) between 08 October 2019 and 30 December 2019.” See *line 169-173 in clean version and 195-198 in track changes version*

Line 129: Kindly use the word participants' instead of the word ‘participant’.

**Response:** Replaced the word “participant” with “participants”

Line 162: Use the word 'made' instead of the word 'make'

**Response:** The word “make” was replaced with “made” as suggested.

Lines 176-177: Something is missing from this sentence. Kindly review and rephrase it.

**Response:** We have added the missing word “were”; to read “Response options on a Likert scale were: ..” see *line 241 in clean version and 304 in track changes version*

Line 188: The use of the word range, there is an expectation to have two values i.e. the

lowest value and the highest value.

**Response:** We have added the maximum value, and the new sentence now reads: “Cronbach alpha and Kaiser-Meyer Olkin (KMO) measure of sampling adequacy (MSA) values for the constructs were all within the acceptable range of 70% to 100%.” See *line 251-252 in clean version and 314-315 in track changes version*

**Discussion**

Lines 278-279: Please state 'in Zimbabwe' at the end of the sentence. I also think that

merging the first and second sentence would read better. i.e. ' We found that 17% of the

participants had never had an HIV test, in rural communities in Zimbabwe between 2018-

2019 despite a community-based distribution of HIV self-testing kits which greatly

improved access to HIV testing'

**Response:** We agree with the suggestion and the 2 sentences were combined. See *line 356-358 in clean version and 442-444 in track changes version*

Line 280: Kindly delete the word 'however' from the sentence. Also replace the word 'the' at

the beginning of the sentence with the word 'these results'

**Response:** We have reworded the sentence to: “These results are comparable with the findings from a household survey done in Zimbabwe in 2020 which reported that 20% and 16% in rural and urban areas respectively had never tested for HIV.” See *line 363 in clean version and 452 in track changes version*

Line 286: These references should be merged i.e. [23-27]

**Response:** References were merged in *line 369 in clean version and 458 in track changes version*

Line 287: This reference is about men in West Africa. Kindly use a reference from the East

and Southern Africa region. J K Chikovore has written a bit on masculinities and health

seeking behaviout in this region

**Response:** We have substituted this with 2 references (*line 369-370 in the clean version and 458-459 in the track changes version)* from Sub-Saharan Africa ie

- *Chikovore J, Hart G, Kumwenda M, Chipungu GA, Desmond N, Corbett L. Control, struggle, and emergent masculinities: a qualitative study of men's care-seeking determinants for chronic cough and tuberculosis symptoms in Blantyre, Malawi. BMC Public Health. 2014;14:1053.*
- *Camlin CS, Ssemmondo E, Chamie G, El Ayadi AM, Kwarisiima D, Sang N, et al. Men "missing" from population-based HIV testing: insights from qualitative research. AIDS Care. 2016;28 Suppl 3(Suppl 3):67-73.*

Lines 293-303. Results shows that 16% of 45+ also never tested. The authors have not

discussed this component in this paragraph.

**Response:** In response to the comment, we have added the following lines: “We also found that individuals aged 45 years and above, had higher odds of being never testers compared to 35-44 years age group. This is likely attributed to low HIV risk perception among the older age group as reported in this study. Studies among young people have reported a positive correlation between HIV testing uptake and risk perception [48, 49]. However, the reason why those at least 45 years were unlikely to test for HIV needs to be investigated further.” See *line 386-391 in clean version and 475-480 in track changes version*

Line 315-316: I suggest leaving the reasons why unemployed were unlikely to self-test as a

research gap that needs qualitative research to investigate.

**Response:** Thank you for the suggestion. Since we did not have a reference, we replaced the sentence “Therefore, we believe that the reason why the unemployed were unlikely to test was other priorities.” with “Therefore, further investigations are needed to understand the reasons why the unemployed were less likely to test.” as suggested. See *line 402-404 in clean version and 492-495 in track changes version*

Lines 322-323: Can you support this statement with references on the relationship

between perceived low risk of contracting HIV and non-uptake of HIV testing.

**Response:** We added the following references, *line 390 in clean version and 479 in track changes version*;

- *Dzinamarira Tafadzwa, Enos M. Adolescents and young people in sub-Saharan Africa: overcoming challenges and seizing opportunities to achieve HIV epidemic control. Front Public Health. 2024;12.*
- *Ajayi AI, Mudefi E, Adeniyi OV, Goon DT. Achieving the first of the Joint United Nations Programme on HIV/AIDS (UNAIDS) 90-90-90 targets: understanding the influence of HIV risk perceptions, knowing one's partner's status and discussion of HIV/sexually transmitted infections with a sexual partner on uptake of HIV testing. Int Health. 2019;11(6):425-31*.

Lines 330-332: The International AIDS Society has done some fantastic work in the area of

HIV stigma 'Getting to the Heart of Stigma'. They do have great references on how to

address stigma in the context of HIV. Kindly explore if you can use some of their

references. See: https://www.iasociety.org/sites/default/files/JIAS/JIAS_Vol25-

S1_complete_file.pdf

Response: Thank you for sharing the link. We have added the following text at lines *line 420-428 in clean version and 511-519 in track changes version*:

“In addition, other ways to reduce HIV related stigma and discrimination include, but is not limited to: strengthening capacity of community health workers by ensuring appropriate linkages between communities and formal health systems; ensuring that young people have access to youth-friendly HIV services and comprehensive sexuality education; engaging communities in stigma and discrimination-reduction activities; educating workplace communities on HIV, comorbidities; legal literacy to promote positive social norms related to HIV; and routinely assessing knowledge, attitudes and practices of health-care workers towards vulnerable populations to support health facility administrators to identify and address any issues[61].

**References**

Line 482-483: Reference is quite old. Kindly use more recent papers

**Response:** Thank you for noting that, we have added this more recent reference in *line 415 in clean version and 506 in track changes version*

- *Mahlalela NB, Manne-Goehler J, Ohene-Kwofie D, L BA, Montana L, Kahn K, et al. The Association Between HIV-Related Stigma and the Uptake of HIV Testing and ART Among Older Adults in Rural South Africa: Findings from the HAALSI Cohort Study. AIDS Behav. 2024;28(3):1104-21.*

**Reviewer #2**: Overall, this work appears to highlight key demographic variables that are

predictive of whether an individual tests for HIV. However more detail is needed to explain

and justify statistical methodology and further interpretation of key results including

reporting point estimates/p-values/confidence intervals in the main text would be helpful.

Authors mention the frequency of missing data was less than 1.5% for all variables, except

one with 5% missing. Overall, authors should report the total percent of missing

observations that were excluded from analyses. And were these observations excluded

only in the analyses for which the missing variable was included in the model? Or were

observations with any missingness dropped entirely from all analysis? This needs to be

clarified.

**Response:** We have now reported missingness by variable in Table 1 on descriptive characteristics and clarified how participants with missing data were treated in the analysis. We also had erroneously stated that only one variable had 5% missingness. To address this, we added the following sentences in the Methods (*line 194-199 in clean version and 226-231 in track changes version*): “Six variables used in our analysis had missing data, and the frequency of missing or “declined to answer” combined was low: perceived health status (1.0%), employment status (1.1%), religion (1.4%), steady partner status (1.7%), “treatment optimism” (5.0%) and wealth quintile (5.1%). Individuals with missing data were excluded only from the analyses in which the respective missing variable was included when investigating factors associated with never having tested for HIV (ie in the regressions).”

For the principal component analysis, it would be helpful to report the percent of the

variance that is explained by the first principal component which is included in analyses.

This allows us to know how much information is lost by using the single summary measure

rather than the individual items.

**Response:** This information is included in the accompanying supplementary document; *S2 Table Derivation of constructs*. However, we have added the following sentence in the manuscript at lines *line 234-236 in clean version and 297-299 in track changes version*, for community cohesion: “The variance explained by the first factors of the sub-construct variables was as follows: 64% for social cohesion, 58% for shared concern, and 66% for “critical consciousness”; at lines *244-246 in clean version and 307-309 in track changes version*, for stigma: ”The variance explained by the first factors of sub-construct variables was, 70% for stigma (negative attitudes), and 53% for perceived stigma in the community” and at *line 249-250 in clean version and 312-313 in track changes version*, for treatment optimism, “The Cronbach alpha was 75%, with the first factor explaining 58% of the variance. ”

It is unclear why the authors choose to create quantile cutoffs and categorize the resulting

scores from the PCA analyses for the wealth, participation in decisions, community

cohesion, stigma, treatment optimism explanatory variables. Why not use the continuous

scores obtained from PCA? If this decision was done to reflect previous literature, it should

be stated.

**Response:** The primary manuscript which used data from this CRT used categorised wealth scores from polychoric PCA. We replicated how the variable was generated, using the same analysis code. In addition, categorised scores have been previously used in demographic and health surveys.

For community cohesion, stigma, treatment optimism explanatory variables, we categorised the scores in tertiles as we felt the approach makes the interpretation intuitive to the readers. We understand that aggregating scores may lead to loss of granularity and other important information about the variables. However, we believed grouping the scores would enhance clarity of our findings, particularly those who might not be familiar with PCA scores, thus improving accessibility of our results.

For “participation in decisions”, the variable was generated using three questions on decisions about major household purchases, visiting family members and health care as explained in *line 210-215 in clean version and 244-249 in track changes version*. We decided to create a new variable “number of decisions” as we felt it will be easier for the reader to understand.

Overall, the section labeled ‘Construct explanatory variables’ was confusing to read. I

would recommend first outlining the procedure (PCA, then division of scores into

categories) that was used across all the constructed variables, and then provide details on

the summarization of each distinct measure.

**Response:** As recommended by the reviewers we revised the section. This paragraph (*line 216-265 in clean version and 278-318 in track changes version*) now reads as follows: “Factor analysis, a variable reduction technique with one factor solution using the principal component factoring (PCF) method was conducted on community cohesion subscales (social cohesion, shared concern, and critical consciousness), stigma scales (“perceived stigma in community”, “stigma (any negative attitudes)”, and “HIV attitude and treatment optimism)” scales. The factor solution was then rotated using the promax method.

The factor scores for community cohesion subscales and “perceived stigma in community” scales were then summarised at a cluster level using the median, and finally the clusters were divided in tertiles and each cluster labelled as either low, medium or high based on the calculated median. The “stigma (any negative attitude)” and “HIV attitude treatment optimism” scales scores were divided into tertiles at individual level and each individual was labelled respectively as either having low, medium or high stigma and or low, medium or high HIV attitude and treatment optimism.

A total of 27 questions were related to community cohesion (See S2 Table). These were grouped into three subscales: social cohesion (6 items), shared concern (10 items) and critical consciousness (11 items). Response options for the items were: strongly agree, somewhat agree, neither agree nor disagree, somewhat disagree, and strongly disagree. The internal consistencies of the subscales as measured by Cronbach alpha were 88% (social cohesion), 92% (shared concern) and 95% (critical consciousness). The community cohesion questions were adopted from the Lipman Study [26]. The variance explained by the first factors of the sub-construct variables was as follows: 64% for social cohesion, 58% for shared concern, and 66% for “critical consciousness”.

Ten questions related to stigma (see S2 Table) were adapted from the Hargreaves paper and had previously been validated [27]; they were grouped into 3 subscales: “stigma in health care settings” (2 items), “perceived stigma in community” (5 items) and “stigma: any negative attitude (fear and judgement)” (3 items). Response options on a Likert scale were strongly agree, agree, unsure, disagree, and strongly disagree. Cronbach alpha for the sub-scales were 66% (stigma in healthcare settings), 78% (“perceived stigma in the community”) and 78% (any negative attitude). The variance explained by the first factors of the sub-construct variables was, 70% for “stigma (any negative attitudes)” and 53% for “perceived stigma in the community”.

The “treatment optimism” variable was measured through four questions obtained from WHO generic tools for operations research[28] (see S2 Table), rated on the same 5-point Likert scale as the questions related to stigma. The Cronbach alpha was 75%, with the first factor explaining 58% of the variance.

Cronbach alpha and Kaiser-Meyer Olkin (KMO) measure of sampling adequacy (MSA) values for the constructs were all within the acceptable range of 70% to 100% [29-31], except for “stigma in health care settings” where both the Cronbach alpha (66%) and the KMO MSA (50%) fell below the acceptable range. As a result, “stigma in health care settings” was excluded from the analysis.”

On line 206 need to state how mixed effect model was specified (mixed effects by cluster?

Random intercept only?)

**Response:** Thank you for the input. We have added “with a random intercept at headman unit level (to account for clustering within each headman unit)”, and the new sentence now reads;” Unadjusted and adjusted mixed effects logistic regression with a random intercept at headman unit level (to account for clustering within each headman unit) was used to model the relationship between independent variables and the outcome”. *line 272-274 in clean version and 336-337 in track changes version*

Authors should explain why the two adjustment sets (sex and age vs sex, age, religion,

education) were selected and what they expect either model to show.

**Response:** We appreciate the reviewers' insightful comments regarding the two adjustments. “In trying to avoid over adjustment, we initially adjusted for age and sex, the commonly recognised potential confounding variables. We then introduced religion and education to the model as they are known predictors of testing behaviour. After noting that the estimates were comparable, we decided to present all the results, trying to demonstrate transparency and that our potential readers will have clear understanding of all the analyses conducted.”

We also added a statement explaining the 2 adjustments in the methods section.” This strategy was done to reduce the risk of over adjustment. We presented the results from the two models to show the effect of additional adjustment for religion and education.” See *line 277-279 in clean version and 340-344 in track changes version*

Some more discussion might be given to the point on lines 220-222. It seems this might

impact the overall validity of results, as it would seem there is some measurement error in

the outcome. Is there any reason why this might be happening? This should be mentioned

in the limitations in the discussion.

**Response:** We felt that 0.66% (74/11,150) was a relatively small number, and unlikely to have an impact on validity of the results. However, we acknowledge mentioning the inconsistency as a limitation of the study, to show transparency. The inconsistency could be due to the fact that our data collection tool allowed participants to mistakenly indicate they had never been tested for HIV, despite having previously declared of having received a positive result, or erroneously report an HIV-positive status when they had reported never having been tested for HIV. The questionnaire took about an hour to complete, and we suspect some participants might have provided some answers in the hope to finish the questionnaire more quickly

As a limitation we added the following in the manuscript: “Another limitation is the exclusion of (74/11,150) 0.66% participants with inconsistent responses. However, the proportion is relatively small such that it is unlikely to have an impact on the validity of the results.” See *line 442-444 in clean version and 534-537 in track changes version*

In Table 1, how are the p-values obtained? Does this analysis account for the correlation by

cluster that is present in the data? If not, these results are not valid and should not be

reported. If it does, this analysis should be equivalent to the unadjusted mixed effects

model and would be repetitive to report in both tables.

**Response:** Thank you for the observation. We removed the p values in Table 1, as they did not account for the correlation by cluster and as the reviewer mentions they would be equivalent anyway.

For all models, the choice of reference groups is confusing and inconsistent. For example,

selecting age 34-44 as the reference group and comparing to those older and younger is

unusual, as typically the reference is selected as the most extreme category (either

youngest or oldest, in this example). For the constructed variables, it would make sense to

select consistently across all either low or high as the reference. The selection of reference

groups here feel a bit like the authors might be arbitrarily searching for significant results.

**Response:** We acknowledge that reference groups are usually chosen as the most extreme categories. However, we found it clearer to avoid a double negative interpretation in our results (for OR<1), given that our outcome "Never testing for HIV" conveys a negative aspect. So, reference groups for these categories were selected in such a way that our odds ratios are greater than 1.

Rather than just report the significance in the main text of the results, it would be helpful to

also report p-values and or the effect estimates with confidence intervals to highlight

important findings.

**Response:** We have added estimates and confidence intervals in the main text. Initially we had though that readers will refer to the table of results for the actual odds ratio and confidence intervals.

**Smaller concerns**

Weird/unclear phrasing examples

o Line 146 “Also, below is a summary of how certain variables were derived.”

**Response:** This is well noted, the sentence was rephrased to: The next paragraph gives a summary of how certain variables were derived. See *line 193-194 in clean version and 222-223 in track changes version*

o Line 156-157 “and took scores for the first principal component as household scores”

**Response:** We rephrased the statement to “and then derived household scores based on the first principal component” See *line 207-208 in clean version and 240-241 in track changes version*

o Line 354, interpretation should be: males, age below above … were more likely to be

never testers

**Response:** The passage was revised.

Would recommend using quotes around variable names

o For example, on line 160, write ‘participation in decisions’

**Response:** Thank you for the suggestion**,** Quotes were added.
